# Laparoscopic fenestration of a symptomatic giant splenic cyst in a young adult: A case report

**DOI:** 10.1016/j.ijscr.2025.111632

**Published:** 2025-07-09

**Authors:** Andres Fontaine-Nicola, Patricia Ruiz-Cota, Ryan Broderick

**Affiliations:** a Division of Minimally Invasive Surgery, Department of Surgery, University of California San Diego, San Diego, CA 92093, USA

**Keywords:** Laparoscopic surgery, Fenestration, Spleen, Massive cyst

## Abstract

**Introduction:**

Splenic cysts are rare lesions typically discovered incidentally through imaging. While many are asymptomatic, large or symptomatic cysts may require surgical intervention to avoid complications. Advances in surgical technique and recognition of splenic function have shifted treatment from routine splenectomy toward organ-preserving approaches.

**Presentation of case:**

A 25-year-old male presented with early satiety, nausea, intermittent vomiting, and persistent left shoulder pain. Imaging revealed a 15 cm unilocular splenic cyst incidentally discovered during a Computed Tomographic Angiography (CTA) for asthma exacerbation. Magnetic Resonance Imaging (MRI) findings favored a chronic hematoma or epithelial cyst. Interventional radiology (IR)–guided aspiration ruled out malignancy or infection. Due to persistent symptoms and lesion size, the patient underwent laparoscopic fenestration. Pathology confirmed a benign fibrous cyst wall and splenic debris. The postoperative course was uneventful, and the patient remained asymptomatic at follow-up.

**Discussion:**

Nonparasitic splenic cysts are rare and often incidentally discovered. While frequently asymptomatic, large cysts can cause nonspecific symptoms and present diagnostic challenges. Imaging and aspiration may guide management, but definitive surgical intervention is often required due to recurrence risk and inconclusive findings. Laparoscopic fenestration allows for effective treatment while preserving splenic function, minimizing morbidity, and long-term immunologic risks.

**Conclusion:**

Laparoscopic fenestration is a safe and effective treatment for symptomatic giant splenic cysts. In appropriately selected patients, spleen preservation can be achieved with favorable outcomes and minimal complications.

## Highlights

• Rare case of a giant splenic cyst discovered incidentally on CTA.
• Symptomatic presentation with shoulder pain, satiety, and nausea.
• MRI supported a differential including chronic hematoma or pseudocyst.
• Successful laparoscopic fenestration with spleen preservation.
• Multidisciplinary evaluation guided surgical management.

## 1 Introduction

Splenic cysts are uncommon lesions, often asymptomatic and incidentally found. Cysts are typically classified as true cysts or pseudocysts. True cysts can be parasitic or nonparasitic in origin, whereas most pseudocysts are a result of previous trauma [1]. The prevalence of splenic cysts has increased secondary to the widespread use of abdominal imaging and successful nonoperative management of traumatic splenic injuries. Treatment previously consisted primarily of total splenectomy. However, recognition of the importance of the spleen throughout a patient's life has led to changes in the management of splenic disease [2]. Even so, when they are large or symptomatic, surgical intervention is warranted to prevent complications. This work has been reported in line with the SCARE guidelines criteria [3].

## 2 Presentation of case

**Fig. 1a-b:**
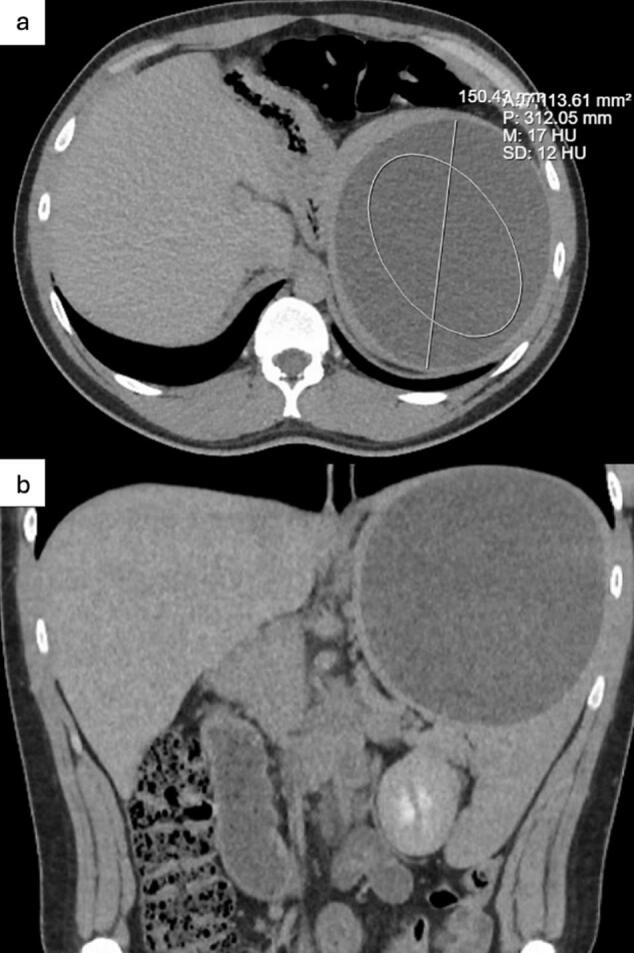
Contrast-enhanced Computed Tomography Angiography (CTA) of the abdomen incidentally revealing a large, hypoattenuating splenic lesion measuring approximately 15 cm in maximal diameter.

**Fig. 2:**
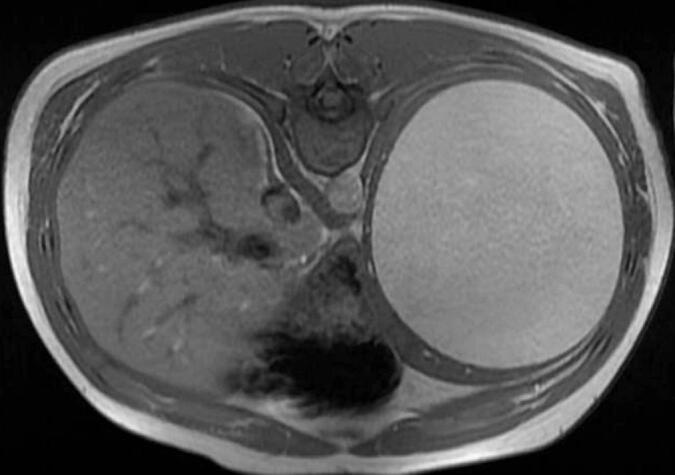
Magnetic Resonance Imaging (MRI) of the abdomen showing a unilocular cystic lesion within the spleen measuring 14.8 × 13.5 × 14.1 cm with high T1 signal intensity.

A 25-year-old male presented with a massive 15 cm splenic cyst incidentally discovered during CTA for asthma exacerbation and pulmonary embolism (PE) suspicion, which was excluded (Fig. 1a-b). MRI revealed a complex cystic lesion (Fig. 2). He reported early satiety, bloating, nausea, intermittent vomiting, and persistent left shoulder pain. IR–guided aspiration showed no infection or malignancy. After multidisciplinary discussion, laparoscopic fenestration with spleen preservation was the therapeutic decision.

The patient was placed onto the operating room table. Pneumatic compression stockings were placed on both lower extremities and then the patient underwent induction of general endotracheal anesthesia. An orogastric tube was then placed. Intravenous antibiotics were administered.

The patient was then rolled into the right lateral decubitus position with an axillary role placed under the right axilla. Both the arms and the legs were cushioned appropriately to protect all joints and pressure points. The operating room table was maximally flexed to open up the patient's costovertebral angle. The table was rolled far to the left and the security of the patient's position on the operating room table was assured. Once this was established, the abdomen was clipped and then the abdomen, groins, left flank and back were prepped widely with chlorhexidine and then draped sterile.

The first trocar was placed 5 cm to the left of the umbilicus. Under direct view with the camera system, the abdomen was entered. Intraabdominal pressure was raised to 15 mmHg with CO₂. Two more trocars were placed, one 5 mm in the left flank and one 15 mm in the left subcostal space.

**Fig. 3:**
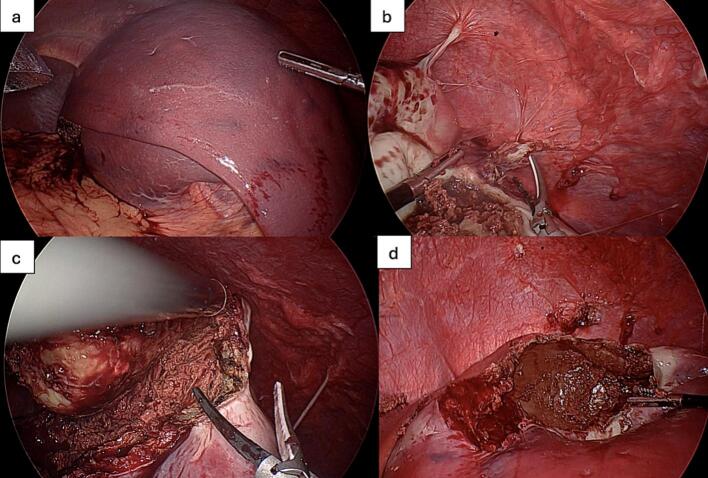
Intraoperative images during laparoscopic fenestration. **(a)** Initial view of the massive spleen with the superior border of the cyst exposed. **(b)** Adhesions between the abdominal wall and the cyst wall being dissected with the harmonic scalpel. **(c)** Cyst cavity decompression after puncture with harmonic scalpel; brown serous fluid begins to evacuate. **(d)** Empty cyst after evacuation of over 1.2 l of thin, brown fluid using a laparoscopic suction-irrigation system. (For interpretation of the references to colour in this figure legend, the reader is referred to the web version of this article.)

Intraoperatively, the spleen was giant (Fig. 3a), and the superior border of the cyst could be seen. There were adhesions of the abdominal wall to the splenic cyst, which were taken down with a harmonic scalpel (Fig. 3b). The inferior edge of the cyst wall, bordering normal spleen, was identified. The harmonic scalpel was used to puncture through the wall into the cyst cavity, where spleen parenchyma and copious thin brown fluid were encountered (Fig. 3c). This was suctioned and controlled with a laparoscopic suction-irrigator, removing over 1.2 l of fluid (Fig. 3d).

The cyst wall was then divided circumferentially where it met normal spleen and sent for pathology. Additional adhesions to the left diaphragm were carefully lysed, protecting the diaphragm. More cyst wall was excised, placed in a second bag, and sent for pathology. Near-total excision of the anterior wall was performed, with the residual cavity expected to fibrose.

The cyst contents consisted of brown, friable, sponge-like tissue. Roughly 1 l of this tissue was removed. A portion was bluntly sampled and sent for separate pathology analysis. Tumor markers (CEA and CA 19–9) and cytology were obtained via IR-guided aspiration and were negative, helping to exclude malignancy. The pathologic report revealed a splenic cyst wall. Fragments of spleen parenchyma with attached fibrous cyst wall with amorphous material, including fibrin deposits and cholesterol clefts, were found. There was no malignant tissue in the specimen and no epithelial lining, consistent with a pseudocyst.

Multiple oozing points along the spleen edge were managed with an argon beam coagulator and surgical powder. Argon beam was used for broad, uniform coagulation on fragile splenic surfaces. Hemostasis was excellent, so no drain was placed. Hemoglobin levels dropped from 13.9 to 13.6 g/dl postoperatively. No transfusion was required.

Due to his asthma background, preoperative optimization included inhaled bronchodilators. No systemic steroids were required. The postoperative course was uneventful with standard respiratory management. The postoperative course was uneventful. The patient resumed a regular diet, required only oral analgesia, and was discharged on postoperative day two. At one-month follow-up, he remained asymptomatic with no evidence of recurrence. He continues to do well under routine surveillance. The pneumococcal vaccine was discussed but not administered since the spleen was preserved.

## 3 Discussion

Nonparasitic splenic cysts are uncommon and are frequently discovered incidentally during imaging for unrelated complaints [4]. They may remain asymptomatic or present with vague symptoms such as bloating, early satiety, or referred shoulder pain due to diaphragmatic irritation. When large, these lesions may pose diagnostic and therapeutic challenges, particularly in differentiating benign cysts from other splenic masses, including pseudocysts, hematomas, hydatid cysts, and cystic neoplasms [5].

In this case, the lesion was first identified incidentally on CTA, where vascular mapping excluded significant vascular involvement. Later, the lesion was further characterized by MRI, which revealed a large unilocular cyst with high T1 signal. While the imaging findings favored a benign etiology, the lesion's size, progressive symptoms, and inconclusive aspiration prompted surgical management. Aspiration alone carries a high recurrence rate and limited diagnostic yield, especially for nonparasitic epithelial cysts. Thus, definitive surgical intervention was indicated.

Laparoscopic fenestration was chosen to manage the lesion while preserving splenic tissue, consistent with current recommendations favoring spleen-preserving techniques whenever feasible [[6], [7], [8]]. Splenectomy, while curative, carries increased risks of postoperative infection and long-term immunologic consequences, especially in young patients. Another complication with splenectomy is bleeding, so this is another reason to choose fenestration if possible. In this case, minimally invasive fenestration resulted in rapid symptom resolution, no complications, and no evidence of recurrence on follow-up. We should acknowledge that one month of follow-up is not enough to assess recurrence, so follow-up with imaging is essential to be scheduled at 3, 6, 12, up to 24 months, ideally if the patient remains asymptomatic. An excellent point to take into consideration is to keep in mind the low recurrence that this approach has, as it has been reported in previous studies [9,10].

This case reinforces the importance of considering splenic cysts in the differential diagnosis of nonspecific upper abdominal symptoms and highlights the role of laparoscopic fenestration as a safe, effective, and organ-sparing approach in appropriately selected patients [8].

## 4 Conclusion

This case illustrates how large splenic cysts, though rare and often incidental, can cause significant symptoms and diagnostic uncertainty. Minimally invasive spleen-preserving techniques such as laparoscopic fenestration offer a safe and effective treatment option in selected cases. Early multidisciplinary evaluation and tailored surgical planning are essential to optimize outcomes and avoid unnecessary splenectomy.

## Consent

Written informed consent was obtained from the patient for publication and any accompanying images. A copy of the written consent is available for review by the Editor-in-Chief of this journal on request.

## Ethical approval

Ethical approval was not required for this case report, as case reports involving a single patient are deemed not to constitute human subjects research according to institutional policies.

## Patient perspective

The patient expressed satisfaction with the resolution of symptoms and the quick postoperative recovery, highlighting relief from shoulder pain and abdominal discomfort.

## Declaration of Generative AI and AI-assisted technologies in the writing process

During the preparation of this work, the authors did not use artificial intelligence (AI) to assist in language clarity and manuscript organization.

## Funding statement

The authors received no funding or financial support for this study.

## Declaration of competing interest

The authors have no conflict of interest to declare.
